# Activity of Ancillary Heterotrophic Community Members in Anaerobic Methane-Oxidizing Cultures

**DOI:** 10.3389/fmicb.2022.912299

**Published:** 2022-06-02

**Authors:** Qing-Zeng Zhu, Gunter Wegener, Kai-Uwe Hinrichs, Marcus Elvert

**Affiliations:** ^1^MARUM – Center for Marine Environmental Sciences, University of Bremen, Bremen, Germany; ^2^Max Planck Institute for Marine Microbiology, Bremen, Germany; ^3^Faculty of Geosciences, University of Bremen, Bremen, Germany

**Keywords:** anaerobic oxidation of methane, archaea, bacteria, heterotrophy, stable isotope probing, lipid biomarkers

## Abstract

Consortia of anaerobic methanotrophic archaea (ANME) and sulfate-reducing bacteria mediate the anaerobic oxidation of methane (AOM) in marine sediments. However, even sediment-free cultures contain a substantial number of additional microorganisms not directly related to AOM. To track the heterotrophic activity of these community members and their possible relationship with AOM, we amended meso- (37°C) and thermophilic (50°C) AOM cultures (dominated by ANME-1 archaea and their partner bacteria of the Seep-SRB2 clade or *Candidatus* Desulfofervidus auxilii) with L-leucine-3-^13^C (^13^C-leu). Various microbial lipids incorporated the labeled carbon from this amino acid, independent of the presence of methane as an energy source, specifically bacterial fatty acids, such as *iso* and *anteiso*-branched C_15:0_ and C_17:0_, as well as unsaturated C_18:1ω9_ and C_18:1ω7_. In natural methane-rich environments, these bacterial fatty acids are strongly ^13^C-depleted. We, therefore, suggest that those fatty acids are produced by ancillary bacteria that grow on ^13^C-depleted necromass or cell exudates/lysates of the AOM core communities. Candidates that likely benefit from AOM biomass are heterotrophic bacterial members of the Spirochetes and Anaerolineae—known to produce abundant branched fatty acids and present in all the AOM enrichment cultures. For archaeal lipids, we observed minor ^13^C-incorporation, but still suggesting some ^13^C-leu anabolism. Based on their relatively high abundance in the culture, the most probable archaeal candidates are Bathyarchaeota, Thermoplasmatales, and Lokiarchaeota. The identified heterotrophic bacterial and archaeal ancillary members are likely key players in organic carbon recycling in anoxic marine sediments.

## Introduction

Methane is the most abundant hydrocarbon in marine sediments. The emission of methane from sediments into the water column and eventually the atmosphere is attenuated by the anaerobic oxidation of methane (AOM), which is performed by anaerobic methane-oxidizing archaea (ANME) and sulfate-reducing bacteria consortia (SRB) (Hinrichs et al., 1999; Boetius et al., 2000; Reeburgh, 2007; Wegener et al., 2016). The ANMEs completely oxidize methane to carbon dioxide, and their partner bacteria use the reducing equivalents produced in this reaction for sulfate reduction (Orphan et al., 2001). This exchange likely involves direct electron transfer mediated by cytochromes and nanowires (McGlynn et al., 2015; Wegener et al., 2015). ANME archaea are found in three clades known as ANME-1, ANME-2, and ANME-3 (Orphan et al., 2002; Niemann et al., 2006). Psychro- and mesophilic ANMEs form a consortium with SRB of the *Desulfosarcina/Desulfococcus* (DSS), classified as Seep-SRB1, or *Desulfobulbus* group (Boetius et al., 2000; Michaelis et al., 2002; Niemann et al., 2006). The thermophilic ANME-1 archaea form a consortium with *Candidatus* Desulfofervidus auxilii (Ca. D. auxilii) in heated sediments (e.g., Guaymas Basin). In contrast to SRBs in the psychro- and mesophilic consortia, *Ca.* D. auxilii has been isolated using molecular hydrogen as an alternative electron donor (Krukenberg et al., 2016).

ANMEs are characterized by diagnostic lipid biomarker patterns. ANME-1 archaea predominantly synthesize glycerol dialkyl glycerol tetraethers (GDGTs) as opposed to ANME-2 and ANME-3 that produce archaeol-based diethers, predominantly hydroxyarchaeol (Blumenberg et al., 2004; Rossel et al., 2008, 2011). Nonetheless, a thermophilic ANME-1 AOM enrichment from the Guaymas Basin revealed a substantial quantity of archaeol lipids in comparison to GDGTs, especially in the active growth phase (Kellermann et al., 2016; Wegener et al., 2016). The corresponding lipid patterns of SRB partners determined from AOM environments and cultures are more diverse and taxonomically only partly distinctive (e.g., Hinrichs et al., 2000; Elvert et al., 2003, 2005; Blumenberg et al., 2004; Niemann and Elvert, 2008). It has been shown that bacterial fatty acids (FAs) from environments dominated by ANME-2 include large proportions of C_16:1ω5*c*_ and cyclopropane (cy)-C_17:0ω5_,_6_ (Elvert et al., 2003; Blumenberg et al., 2004), while those dominated by ANME-1 predominantly produce *ai-*C_15:0_ (Blumenberg et al., 2004; Elvert et al., 2005). All of the aforementioned archaeal or bacterial lipids show strong ^13^C-depletions with δ^13^C values of –70‰ and lower, which are assumed to be caused by the distinctively low δ^13^C values of methane (e.g., Hinrichs et al., 1999; Thiel et al., 1999; Pancost et al., 2001; Orphan et al., 2002; Elvert et al., 2003; Blumenberg et al., 2004).

Multiple stable isotope probing (SIP) experiments indicate that ANMEs and their direct SRB partners predominantly assimilate inorganic carbon (Wegener et al., 2008; Kellermann et al., 2012). Specifically, ANME-1 was classified as a methane-oxidizing chemoorganoautotroph (Kellermann et al., 2012). Here, we used long-term meso- and thermophilic AOM enrichment cultures obtained from hydrocarbon-rich heated sediments in the Guaymas Basin. The mesophilic culture grown at 37°C (AOM37) is dominated by ANME-1 and Seep-SRB2; the thermophilic culture maintained at 50°C (AOM50) is dominated by ANME-1 and *Ca.* D. auxilii (Krukenberg et al., 2016; Wegener et al., 2016). Although maintained for 5 years with methane as the sole energy source, these cultures contain substantial numbers of additional bacteria and archaea (Wegener et al., 2016). The functions and carbon sources of these ancillary microbes and their relationship with the AOM consortia remain largely unknown. Kellermann et al. (2012) suggested that these uncultured microbes may be heterotrophs, which likely feed on labile organic compounds, such as acetate or protein-like dissolved organic carbon detected in the pore waters of AOM environments (Heuer et al., 2006; Yoshinaga et al., 2015; Yang et al., 2020; Hu et al., 2021; Pérez Castro et al., 2021).

Leucine is one of the most abundant amino acids produced by microorganisms and, if released into the environment, becomes a carbon, nitrogen, and energy source (Kirchman et al., 1985). Because leucine metabolism was found to be particularly essential during starvation conditions (Harwood and Canale-Parola, 1981; Mårdén et al., 1987), it is ideal for tracking heterotrophic activity in slow-growing enrichment cultures, such as AOM consortia. To explore the activity of these heterotrophic community members and their signaling lipids in AOM environments, we incubated active Guaymas Basin AOM enrichment cultures with ^13^C-leu, a particular precursor for iso-branched FAs (cf. Aepfler et al., 2019), which are abundant in natural ANME-1 systems. Additionally, we used the same cultures devoid of methane to suppress the activity of AOM consortia members and to track the utilization of leucine for lipid biosynthesis by non-AOM microbes. Based on our ^13^C-leu incubation and published microbial community data on the same cultures (Wegener et al., 2016; Krukenberg et al., 2018), we were able to trace ancillary heterotrophic bacteria and archaea in AOM enrichment cultures, detected by strong ^13^C-enrichments of diagnostic FAs but only minor for archaeal lipids, highlighting the identification of branched fatty acids as indicators of bacterial heterotrophy.

## Materials and Methods

### Anaerobic Oxidation of Methane Cultures

The production and maintenance of the sediment-free AOM cultures from the Guaymas Basin were performed as described before (Wegener et al., 2016; Laso-Pérez et al., 2018). In brief, both AOM37 and AOM50 were incubated with marine sulfate reducer medium supplemented with trace amounts of vitamins (Widdel and Bak, 1992) under a CH_4_:CO_2_ atmosphere (2.5 atm; 90:10) at temperatures of 37°C and 50°C, respectively. The initial concentration of sulfate was 28 mM. The carbon isotopic composition of methane used was −35‰ (Wegener et al., 2021), and sulfide concentrations were measured as described before (Cord-Ruwisch, 1985). When sulfide concentrations exceeded 15 mM, microbial biomass was transferred into a fresh medium. Under these conditions, the AOM37 and AOM50 cultures show doubling times of 69 and 55 days, respectively (Holler et al., 2011). The sulfate reducer *Ca.* D. auxilii was isolated from AOM50 with hydrogen as the sole electron donor and sulfate as an electron acceptor. It is chemolithoautotrophic and grows at temperatures between 50 and 70°C, and has a doubling time of 4–6 days (Krukenberg et al., 2016).

### Experimental Setup

For all experiments with the AOM cultures, the culture medium was exchanged, and cultures were equally distributed in 156 ml cultivation bottles. In the case of *Ca.* D. auxilii, new dilutions were prepared (5 ml of active culture for inoculation). ^13^C-leu was dissolved in Milli-Q water and sterilized by filtration (Minisart High Flow, PES, 28 mm, 0.1 μm, sterile). The AOM37 and AOM50 cultures were amended with 100 μM of sterilized ^13^C-leu and incubated under different experimental conditions for 28 days (Table 1): experiment 1 with CH_4_ and ^13^C-leu to track characteristic lipid production by microbial community members involved in leucine metabolism during active AOM; experiment 2 with CH_4_ and without ^13^C-leu as a negative control; experiment 3 with ^13^C-leu and without CH_4_ as a positive control to specifically track ancillary community members and identify their lipids by suppressing the activity of AOM consortia. Through these three experiments, we were able to target ancillary microbial communities existing in the current AOM cultures and constrain their potential heterotrophic capabilities. In contrast, experiment 4 utilized the autotrophic *Ca.* D. auxilii culture and was likewise amended with 100 μM of sterilized ^13^C-leu. This experiment lasted for 40 days, and it thoroughly tested whether the partner bacterium *Ca.* D. auxilii can metabolize ^13^C-leu and constrain its lipid pattern. A ^13^C-leu concentration of 100 μM was chosen to ensure a sufficient supply of substrate and to maximize the potential to observe various pathways of leucine metabolism during prolonged incubation, even though the concentration is higher than existing leucine data from estuarine pore water (up to 3 μM, Henrichs and Farrington, 1979).

**TABLE 1:** Overview of incubation experiments.

Enrichment/culture	δ^13^C_*DIC*_ at T_0_	Incubation time (days)	Experiment 1	Experiment 2 negative control	Experiment 3 positive control	Experiment 4
	
			+ CH_4_ + ^13^C-leu	+ CH_4_	+ ^13^C-leu	+ H_2_ + ^13^C-leu
AOM37	−14.9	0–28*	2	2	2	
AOM50	−25.8	28	2	2	2	
*Ca*. D. auxilii	−17.5	40				2

CH_4_ is provided as an energy source in AOM37 and AOM50, while Ca. D. auxilii uses hydrogen (H_2_) as an energy source. *AOM37 was incubated for 0, 0.5, 3, 7, 14, and 28 days, and both biomass and medium in each bottle were harvested to track ^13^C-incorporation into membrane lipids. The numbers indicate the number of bottles used for each experiment.

### Determination of Sulfide Concentration and Isotopic Composition of Dissolved Inorganic Carbon

Sulfide concentrations were used to monitor the growth of AOM consortia and *Ca.* D. auxilii. The medium subsampling of AOM37 for sulfide concentrations analysis was at 0, 0.5, 3, 7, 14, 21, and 28 days; AOM50 medium subsampling was at 0, 7, 21, and 28 days; and *Ca.* D. auxilii medium subsampling was at 0 and 40 days. The subsampling for the measurements of the carbon isotopic composition of dissolved inorganic carbon (δ^13^C_*DIC*_) was performed on the same days to constrain the leucine mineralization (Aepfler et al., 2019). In brief, 1 ml of the sample was taken by syringe from the incubation serum bottles and filtered through a 0.2 μm filter (Minisart regenerated cellulose syringe filter, 15 mm) to remove cells and other particles. Finally, samples were acidified with 100 μl phosphoric acid overnight in an Exetainer vial pre-purged with CO_2_-free air before isotopic analysis. All samples were measured with a Thermo Scientific Delta Ray isotope ratio infrared spectrometer with an analytical error of ±1‰, which is obtained by repeated measurement of the laboratory CO_2_ reference gas (*n* = 8). All isotopic values are reported in the delta notation as δ^13^C relative to the Vienna PeeDee Belemnite (VPDB) standard.

### Lipid Extraction, Identification, Quantification, and Isotopic Analysis

Due to potential contamination, we avoided subsampling for lipid analysis from the same bottle as used for sulfide concentration and δ^13^C_*DIC*_ determination by obtaining biomass from replicate samples. Cell pellets from these incubations were extracted wet using a modified Bligh and Dyer protocol (Sturt et al., 2004). Before extraction, 1 μg of 1,2-diheneicosanoyl-*sn*-glycero-3-phosphocholine and 2-methyloctadecanoic acid were added as internal standards. Polar lipid-derived fatty acids (PLFAs) in the total lipid extract (TLE) were converted to fatty acid methyl esters (FAMEs) using saponification with KOH/MeOH and derivatization with BF_3_/MeOH (Elvert et al., 2003). Archaeal intact polar lipids (IPLs) in the TLE were separated from the apolar core lipids (CLs) using preparative liquid chromatography (Meador et al., 2015), followed by ether cleavage of both fractions with BBr_3_ in dichloromethane and reduction of the resulting alkyl bromides with superhydride to form isoprenoid hydrocarbons (Jahn et al., 2004). The hydrocarbon products were purified by silica gel column chromatography using 4 ml of hexane as an eluent. Both FAMEs and isoprenoid hydrocarbons were measured by gas chromatography coupled to flame ionization detection (GC-FID, Thermo Finnigan Trace GC) for quantification and gas chromatography-mass spectrometry (GC-MS, Trace GC coupled to Trace MS, both from Thermo Finnigan) for structural identification using the protocols described by Aepfler et al. (2019). Using the same GC conditions, lipid δ^13^C values were determined by GC-isotope ratio-MS (Thermo Finnigan Trace GC coupled to a Thermo Scientific Delta V Plus) connected *via* a GC IsoLink interface and are reported relative to VPDB. The precision of a lab FA standard (2-methyloctadecanoic acid, *n* = 3) was greater than 0.7‰, while the deviations of duplicate isotopic measurement of sample FAs were between ± 1‰ and ± 100‰ (for PLFAs with label uptake of >1,000‰).

The incorporation of ^13^C-leu into bacterial lipids expressed as a percentage of ^13^C incorporation was calculated as the product of excess ^13^C and the amount of FA carbon based on the quantification *via* GC-FID. Excess ^13^C is derived from the difference between the fractional abundance (F) of ^13^C in FAs after 28 days relative to T_0_ with F = ^13^C/(^13^C + ^12^C) = R/(R + 1) and R being derived from the measured δ^13^C values as R = (δ^13^C/1,000 + 1) × R_*VPDB*_.

## Results

### Contents and δ^13^C Values of Microbial Lipids in the Original Cultures

**FIGURE 1:**
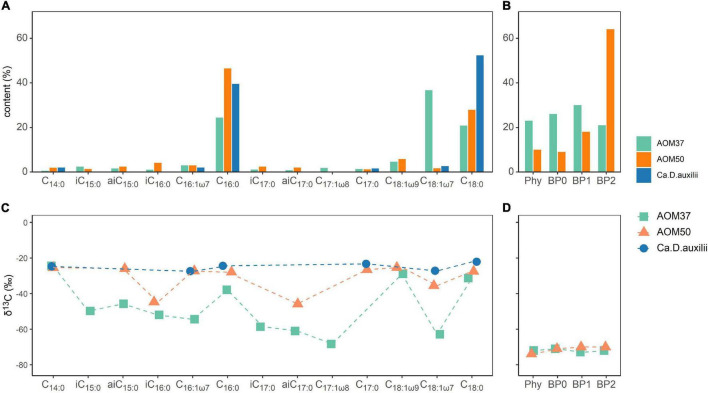
Bacterial and archaeal lipid patterns **(A,B)** and their δ^13^C values **(C,D)** in the original cultures AOM37 and AOM50 and the isolated bacterium *Ca*. D. auxilii. Phy and BP data are derived from total lipid extracts.

The FA distribution in the original AOM37 culture mainly consisted of C_18:1ω7_ (37%), C_16:0_ (24%), and C_18:0_ (21%) (Figure 1A). Branched-chain FAs accounted for 7% of the total. The AOM50 culture was dominated by C_16:0_ and C_18_._0_ with a content of 46 and 28%, respectively. Branched-chain FAs accounted for 12%. The original *Ca.* D. auxilii culture had a FA pattern similar to AOM50, with C_16:0_ (40%) and C_18:0_ (52%) as the dominant FAs. Branched-chain FAs were below the detection limit in the *Ca.* D. auxilii culture. For archaea, we reported the relative content of phytane (Phy) and the three biphytanes (BP0, BP1, and BP2) derived from archaeols and GDGTs, respectively (Figure 1B). The content of Phy in AOM 37 was 23%, higher than that in AOM50 (10%). BPs had similar content in AOM37, with BP1 being highest at 30%. At the higher incubation temperature in AOM50, the BP pattern strongly shifted to BP2 (64%).

In AOM37, the δ^13^C values of monounsaturated FAs ranged from −55 to −68‰ except C_18:1ω9_ with a δ^13^C value of −29‰. Saturated C_14:0_, C_16:0_, and C_18:0_ FAs had less negative δ^13^C values between −25 and −38‰ (Figure 1C). The branched-chain FAs were more ^13^C-depleted, with δ^13^C values ranging from −46 to −61‰. FAs in AOM50 are generally less depleted in ^13^C than AOM37 and showed δ^13^C values between −25 and −45‰, with the most negative δ^13^C values found for the branched-chain FAs *i*C_16:0_ and *ai*C_17:0_. Different carbon fixation pathways of the respective partner bacterium may cause the difference in δ^13^C values of FAs in AOM37 and AOM50. The Seep-SRB-2 partner fixes carbon *via* the Wood-Ljungdahl pathway with a fractionation up to 36‰ (Preuß et al., 1989; Krukenberg et al., 2018), whereas *Ca.* D. auxilii uses the rTCA pathway with a lower carbon isotope fractionation of up to 12‰ (Wirsen et al., 2002; Suzuki et al., 2005; Krukenberg et al., 2016). The δ^13^C values of FAs in the culture of *Ca.* D. auxilii were even more positive, ranging between −21 and −28‰. δ^13^C values of TLE-derived Phy and BPs in the AOM37 and AOM50 were similar and around −70‰ (Figure 1D).

### Temporal Development of Sulfide Production and δ^13^C_*DIC*_ Values During Incubation

**FIGURE 2:**
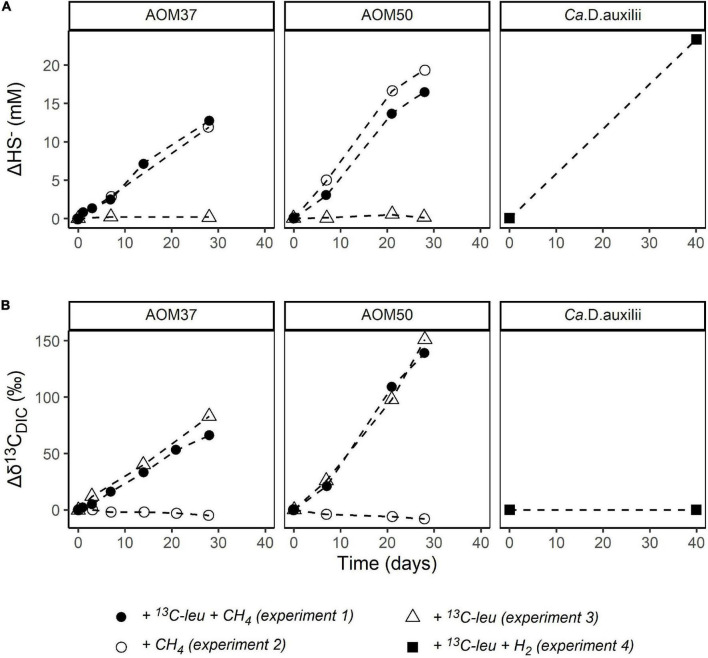
Development of ΔHS^–^ (mM, **A**) and Δδ^13^C_*DIC*_ (‰, **B**) relative to T_0_ in the incubation experiments with the AOM37 and AOM50 cultures over 28 days and the *Ca.* D. auxilii culture over 40 days.

Sulfide concentrations (HS^–^) were measured to monitor the metabolic activity of the microorganisms involved in AOM and of *Ca.* D. auxilii (Figure 2A). In AOM37 and AOM50 cultures without CH_4_ (experiment 3), HS^–^ concentrations remained stable, indicating a lack of methane-dependent sulfate reduction. When CH_4_ was provided (experiments 1 and 2), HS^–^ increased gradually from 2.8 to 15.5 mM (ΔHS^–^ = 12.7 mM) for AOM37 and from 2.2 to 24.7 mM (ΔHS^–^ = 22.5 mM) for AOM50 within 28 days of incubation. There was no substantial difference between incubations with and without ^13^C-leu addition, indicating that leucine did not affect sulfate reduction. For *Ca.* D. auxilii, HS^–^ increased from 2.7 to 26.1 mM (ΔHS^–^ = 23.4 mM) after 40 days of incubation with hydrogen and ^13^C-leu (experiment 4).

We also measured the development of δ^13^C_*DIC*_ values as an indicator of microbial oxidation of ^13^C-leu (Figure 2B). In AOM37 and AOM50 incubated with CH_4_ (experiment 2), δ^13^C_*DIC*_ values decreased from −15 to −20‰ (Δδ^13^C_*DIC*_ = −5‰) and −26 to −34‰ (Δδ^13^C_*DIC*_ = −8‰), respectively, after 28 days of incubation caused by the oxidation of CH_4_. When both CH_4_ and ^13^C-leu were supplied for 28 days (experiment 1), the δ^13^C_*DIC*_ value increased from −15 to +51‰ (Δδ^13^C_*DIC*_ = 66‰) in AOM37 and from −26 to +118‰ (Δδ^13^C_*DIC*_ = 144‰) in AOM50. If only ^13^C-leu was provided (experiment 3), δ^13^C_*DIC*_ increased slightly more from −15 to +68‰ (Δδ^13^C_*DIC*_ = 83‰) and from −26 to +125‰ (Δδ^13^C_*DIC*_ = 151‰) in AOM37 and AOM50, respectively. The slight offset in Δδ^13^C_*DIC*_ values between experiments 1 and 3 results from the dilution of the DIC signal with DIC derived from the oxidation of unlabeled CH_4_ with a δ^13^C value of −35‰ in the former experiment. The continuous increase of δ^13^C_*DIC*_ values suggested a replete supply of ^13^C-leu during the whole incubation process. During incubation of *Ca.* D. auxilii, the addition of ^13^C-leu (experiment 4) did not alter the δ^13^C_*DIC*_ values.

### Alteration of ^13^C Values of Microbial Lipids in ^13^C-Leu Treatments

**FIGURE 3:**
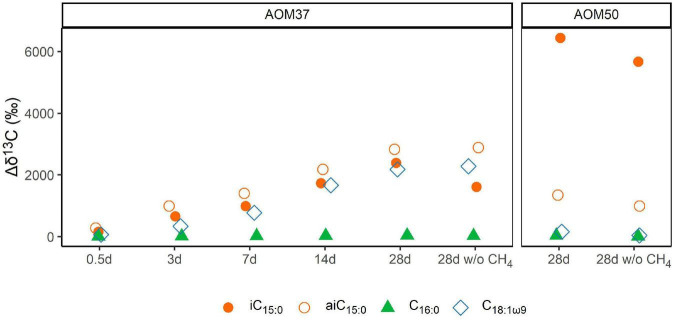
Development of δ^13^C values (in ‰ relative to T_0_) of bacterial FAs during ^13^C-leu incubation of AOM37 and AOM50 with and without (w/o) CH_4_ over 28 days (experiments 1 and 3, respectively).

The lipid compositions of cultures that received ^13^C-leu were similar to those of the original cultures, suggesting that the overall community was stable during the incubations (Figure 1A and Supplementary Table 1), which did not cover a full doubling time. However, the ^13^C-leu additions in experiments 1 and 3 strongly altered the isotopic compositions of bacterial FAs (Figure 3 and Supplementary Table 1). In the AOM37 experiment, the ^13^C-leu addition already resulted in the increase of δ^13^C values by up to 260‰ relative to T_0_ (Δδ^13^C = δ^13^C_*T*_ – δ^13^C_*T*0_) in iso-branched FAs *i*C_15:0_ and *i*C_17:0_ after 0.5 days. After 28 days of incubation, the anteiso-branched *ai*C_15:0_ incorporated most of the ^13^C (δ^13^C = 2,800‰), while *i*C_15:0_ showed a lower value of 2,100‰. In the AOM50 incubation, ^13^C-incorporation was even more pronounced, and δ^13^C values reached up to 6,400‰ for *i*C_15:0_ and *i*C_17:0_ after 28 days. Next to the branched FAs, the monounsaturated FA C_18:1ω9_ was highly labeled in the AOM37 incubation with a δ^13^C value of 2,200‰, which was not the case in AOM50. In both AOM37 and AOM50 enrichment cultures, carbon-numbered saturated FAs were at least ^13^C-labeled. Their δ^13^C values remained lower than those of the DIC, suggesting that the autotrophic partner bacteria mostly synthesize these lipids (Figure 2B). Overall, the δ^13^C values of FAs during incubation without CH_4_ (experiment 3) show a similar ^13^C-labeling strength to those in the incubation with CH_4_ (experiment 1, Supplementary Table 1), indicating that the incorporation of ^13^C-leu is independent of AOM activity. In contrast, the 40-day incubation with ^13^C-leu did not affect the lipid isotopic composition of the isolated autotrophic SRB partner *Ca.* D. auxilii (Supplementary Table 1), confirming its autotrophic lifestyle.

**FIGURE 4:**
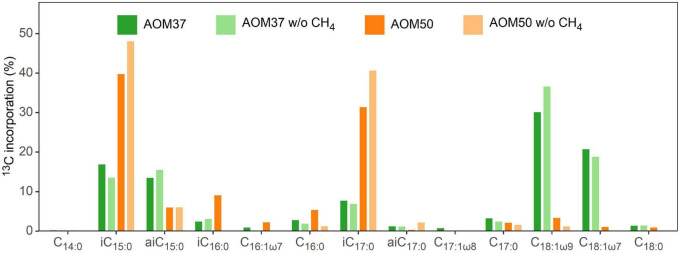
Pattern of ^13^C-incorporation into bacterial FAs in the AOM37 and AOM50 cultures during ^13^C-leu incubation with and without (w/o) CH_4_ after 28 days (experiments 1 and 3, respectively).

We calculated the relative FA ^13^C-incorporation pattern of the heterotrophic bacterial community members of both AOM enrichment cultures (Figure 4) based on the FA content (Figure 1A) and respective δ^13^C values (Supplementary Table 1) after 28 days. In AOM37, the strongest ^13^C-incorporation is observed for the monounsaturated FAs C_18:1ω9_ (30.0%) and C_18:1ω7_ (20.6%), followed by *i*C_15:0_ (16.8%), *ai*C_15:0_ (13.3%), and *i*C_17:0_ (7.5%). In AOM50, we observed the highest ^13^C-incorporation in FAs *i*C_15:0_ (39.6%) and *i*C_17:0_ (31.2%), followed by *i*C_16:0_ (9.0%), while even-numbered FAs (C_16:0_ and C_18:0_) show much less ^13^C-incorporation, despite, as a sum, being the dominant fatty acids in all incubations (Figure 1). These results are independent of whether CH_4_ was supplied to the enrichment cultures or not (Figure 4).

**FIGURE 5:**
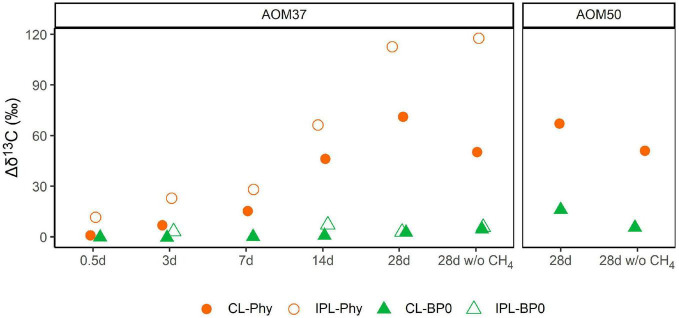
Development of δ^13^C values (in ‰ relative to T_0_) of archaeal lipid-derived isoprenoid hydrocarbons during ^13^C-leu incubation of AOM37 and AOM50 cultures with and without (w/o) CH_4_ over 28 days (experiments 1 and 3, respectively). In the AOM50 culture, the amounts of IPL-Phy and IPL-BP0 were too low to obtain δ^13^C values.

Changes in δ^13^C values relative to T_0_ of CL and IPL derived Phy and BPs in treatments with ^13^C-leu of AOM37 and AOM50 are shown in Figure 5. For all data on archaeal lipid-derived isoprenoid hydrocarbons, we refer to Supplementary Table 2. In both ^13^C-leu experiments, the ^13^C-incorporation into Phy and BPs was lower after 28 days (maximum δ^13^C value of 118‰ in IPL-Phy). These values are in the range of the corresponding ^13^C-label transfer into DIC (δ^13^C_*DIC*_ values up to 151‰; Figure 2). However, the ^13^C-incorporation was independent of AOM activity. Throughout the experiments, BPs incorporated less ^13^C than Phy, regardless of whether they were being retrieved from the CL or IPL fractions. In particular, BP0, which is mostly derived from the GDGT caldarchaeol, had a δ^13^C value up to 16‰ higher in the ^13^C-leu-treated AOM37 and AOM50 culture than in the original cultures, independent of the addition of CH_4_ (experiments 1 and 3 compared to experiment 2). The CL-derived BP1 and BP2, AOM37, and AOM50 did not incorporate the ^13^C-label from ^13^C-leu (Supplementary Table 2). In contrast, CL- and IPL-derived Phy increased by up to 113‰ with CH_4_ (experiment 1) and up to 118‰ without CH_4_ (experiment 3) in AOM37 after 28 days. These values are specifically higher than δ^13^C_*DIC*_ values, which increased by 66‰. For the AOM50 culture, minute amounts of IPL-derived Phy and BPs obtained after ether cleavage prevented isotope analyses.

## Discussion

### Ancillary Microorganisms Grow on Leucine

In the AOM enrichment cultures, sulfide production quantitatively depends on CH_4_ as an energy source. The turnover of ^13^C-leu, as observed by changes in δ^13^C_*DIC*_ values, had no measurable effect on sulfide production (Figure 2A) and occurred independent of the supply of CH_4_, indicating that leucine was predominantly metabolized by ancillary microbes not involved in AOM. This is in line with constant δ^13^C_*DIC*_ values during the 40-day *Ca.* D. auxilii incubation (Figure 2B), providing concrete evidence that *Ca.* D. auxilii does not utilize leucine.

The microbial degradation and assimilation of leucine proceeds in diverse reactions. Leucine is deaminated and decarboxylated, resulting in isovaleryl-CoA, which can be used as a primer for odd-numbered iso-series FAs in bacteria, such as *i*C_15:0_ and *i*C_17:0_ (Kaneda, 1977; Aepfler et al., 2019). In addition, isovaleryl-CoA can be transformed *via* acetoacetate into acetyl-CoA (Yamauchi, 2010; Díaz-Pérez et al., 2016). Acetyl-CoA can either be completely oxidized or used for the synthesis of biomolecules, including the generation of bacterial FAs during elongation *via* malonyl-CoA or isoprenoid ether lipids in the case of halophilic archaea (Harwood and Canale-Parola, 1981; Yamada et al., 2006). Our experiments demonstrated the incorporation of ^13^C-leu into selected branched FAs, such as *i*C_15:0_ and *i*C_17:0_, independent of CH_4_ supply (Figure 4). Additionally, we observed a substantial ^13^C-incorporation into *ai*C_15_ and *ai*C_17_, which is explained by the production of a 2-methylbutyric acid intermediate during leucine catabolism under starvation conditions (Ganesan et al., 2006; Díaz-Pérez et al., 2016). This leads to the formation of 2-methylbutyryl-CoA, which serves as a primer molecule for the synthesis of anteiso FAs. Similarly, the interconversion of leucine and valine gives rise to isobutyryl-CoA (Monticello and Costilow, 1982), which serves as a primer of even-numbered iso-branched FAs, such as *i*C_16_. The high labeling of these fatty acids suggests that abundant heterotrophic bacteria not involved in sulfate-dependent AOM, such as Spirochetes or Anaerolineae (Supplementary Table 3), are generally active and likely to thrive on free amino acids or other protein-like organic matter (Hu et al., 2021). These carbon pools are highly ^13^C-depleted if derived from the biomass of AOM consortia in natural environments (Takano et al., 2018; Hu et al., 2021).

The partner bacterium *Ca.* D. auxilii neither incorporate ^13^C-leu into its dominant FAs C_16:0_ and C_18:0_, nor into any other FAs (Supplementary Table 1). The incorporation of ^13^C-leu differs between the meso- and thermophilic AOM enrichment cultures at 37°C and 50°C, respectively (Figures 3, 4). Compared to AOM37, AOM50 tends to channel more ^13^C from leucine into iso-branched than anteiso-branched FAs (AOM50, iso:anteiso = 92:8; AOM37, iso:anteiso = 63:37). Moreover, in AOM37, we observe a predominant ^13^C-incorporation into straight-chain FAs C_18:1ω9_ and C_18:1ω7_. These straight-chain FAs are likely synthesized by downstream ^13^C-leu products, such as acetate, and are probably derived from ancillary heterotrophic bacterial community members in AOM37 but not in AOM50, such as Spirochetes (Supplementary Table 3).

In the incubation of AOM37 and AOM50 with ^13^C-leu, Phy and BPs (derived from archaeal di- and tetraether lipids, respectively) incorporated much less ^13^C than bacterial FAs (Figures 3, 5). In the AOM37 culture, IPL-derived Phy provided a 47‰ stronger change in its δ^13^C value (Δδ^13^C = 113‰, Figure 5) than the corresponding DIC (Δδ^13^C = 66‰, Figure 2) after 28 days. This divergence suggests that archaeal IPLs are not solely biosynthesized *via* DIC assimilation in AOM37 (Supplementary Figure 1). Eventually, possible carbon substrates are leucine or, more likely, secondary metabolites, such as acetate, which is released during leucine catabolism of heterotrophic bacteria (Aepfler et al., 2019). Unfortunately, we could not examine this relationship in AOM50 due to very few isoprenoid hydrocarbons obtained after ether cleavage. In the ^13^C-leu incubations of our study, the relative ^13^C-enrichment of BPs was negligible compared to Phy but reached up to 16‰ for CL-derived BP0 in AOM50. Based on our short-time incubation, this is in agreement with former labeling experiments of ANME-1 dominated Guaymas Basin sediments using ^13^C_*DIC*_ and D_2_O, which revealed an initial production of diether lipids that are later transformed into tetraether lipids (Kellermann et al., 2016). The observed isotopic evidence of the enhanced formation of ^13^C-enriched archaeal lipids over time suggests that some archaea participated in the leucine metabolism or the assimilation of metabolic intermediates. Nonetheless, their role in amino acid mineralization seems to be less important than heterotrophic bacterial community members due to their low ^13^C-label incorporation.

### Minor Bacterial and Archaeal Community Members Thrive on Anaerobic Oxidation of Methane Necromass

Methane-rich sediments contain a large number of AOM consortia and diverse host archaeal and bacterial communities, with a substantial proportion of heterotrophic microorganisms (Biddle et al., 2006; Ruff et al., 2015; Dombrowski et al., 2018; Pérez Castro et al., 2021). These heterotrophs coexist with AOM consortia in natural environments and enrichment cultures, even after many years of maintenance (Wegener et al., 2016). Prior microbial composition analysis of the Guaymas Basin AOM cultures revealed that ANME-1 archaea and their partners dominate AOM37 and AOM50 (Seep-SRB2 and *Ca*. D. auxilii, Holler et al., 2011; Wegener et al., 2015, 2016). Ancillary microbial communities, identified by amplified 16S rRNA gene sequences of the AOM37 and the AOM50 culture (Wegener et al., 2016) and 16S rRNA genes recruited from the metagenomes of the AOM37 and the AOM60 culture (Supplementary Table 3; Krukenberg et al., 2018), are presumably unrelated to AOM. These include Anaerolineaceae and Spirochetes, and Candidate divisions JS1, WS3, and KB, many of which are known to be heterotrophs. Anaerolineae, which occupy up to 3.7% of the Guaymas Basin cultures (Supplementary Table 3), are strictly anaerobic heterotrophs and thrive on carbohydrates and amino acids (Rosenkranz et al., 2013; Liang et al., 2016). The cultured strains of this group produce mainly iso- and anteiso-C_15_ and C_17_ FAs (Yamada et al., 2006). Spirochetes similarly thrive on the degradation of carbohydrates and proteins (Paster, 2010; Dong et al., 2018) and are abundant in anoxic hydrocarbon-rich habitats (Dong et al., 2018). The AOM37 culture contains up to 5% Spirochetes, whereas these heterotrophs are absent in the AOM50 culture (Supplementary Table 3). Spirochetes primarily synthesize branched fatty acids, but some also produce substantial amounts of C_18:1ω9_ and C_18:1ω7_ (Livermore and Johnson, 1974; Vishnuvardhan Reddy et al., 2013). Hence, in the AOM37 culture, the substantial ^13^C-incorporation into branched fatty acids and C_18:1ω9_ and C_18:1ω7_ FAs is most likely due to the Spirochetes activity (Figure 4). The production of the latter can be attributed to the prolonged transformation and oxidation of isovaleryl-CoA in the tricarboxylic acid cycle, leading to acetyl-CoA and thus the production of even-numbered FAs (Aepfler et al., 2019).

In natural AOM environments, there is circumstantial evidence for the presence of heterotrophic bacteria because of the abundance of branched FAs. Originally, different FA patterns have been described as originating from environments dominated by either ANME-1 or -2 but showing the presence of the same SRB partner (Blumenberg et al., 2004; Elvert et al., 2005; Niemann and Elvert, 2008). ANME-1 dominated AOM systems are related to lower methane flux and are dominated by *ai*C_15:0_ as well as other branched-chain FAs (Stadnitskaia et al., 2008), while ANME-2 dominated systems are indicated by the presence of monounsaturated C_16:1ω5_ and cyC_17:0ω5_,_6_ FAs at sites with high methane flux intensity (Elvert et al., 2003). Taking the results of our study into account, we suggest that the different FA patterns, particularly the larger amounts of branched and partly unsaturated fatty acids, originate from the activity of heterotrophic bacteria inhabiting the vicinity of AOM consortia. These heterotrophic bacteria effectively utilize available amino acids, such as valine, leucine, or isoleucine, which are derived from ^13^C-depleted proteins from AOM consortia necromass or AOM cell exudates/lysates, such as amino acids and acetate (Middelboe and Jørgensen, 2006; Takano et al., 2018; Yang et al., 2020; Hu et al., 2021). Thus, if amino acid-based carbon is available, especially under energy- and nutrient-limited conditions in ANME-1 dominated settings, bacterial heterotrophs will produce more branched FAs. Under natural conditions, such branched FAs become even more negative in δ^13^C values than FAs derived from the autotrophic SRB partner in AOM consortia (Elvert et al., 2005). Moreover, the activity of such heterotrophic bacteria may explain the difficulty of detecting AOM biomarkers in the sulfate methane transition zone (SMTZ) or their disappearance below the current SMTZ (Niemann et al., 2005; Biddle et al., 2006; Zhu et al., 2021) because AOM biomass is more labile and accessible to these degraders than recalcitrant background organic matter. As a result, they actively reshape different carbon pools and contribute to biogeochemical carbon cycling in anoxic marine sediments.

In addition, both AOM enrichment cultures contain archaea with potential heterotrophic metabolisms, including the members of the Bathyarchaeota, Thermoplasmatales, and Lokiarchaeota (Supplementary Table 3; Wegener et al., 2016; Krukenberg et al., 2018). All these three archaeal groups encode protein catabolism or have been cultured on proteinaceous substrates (Imachi et al., 2020; Yin et al., 2022). The metabolic activity of these microbes in the AOM37 culture is supported by the methane-independent incorporation of ^13^C-leu into IPL-derived Phy (Figure 5). Bathyarchaeota—formerly known as the Miscellaneous Crenarchaeotal Group (MCG)—are widespread in anoxic sediments. Based on their genomes, some Bathyarchaeota may be protein-degrading heterotrophs with acetyl-CoA centralized pathways for energy conservation (Lloyd et al., 2013). In the AOM50 culture, Bathyarchaeota, which accounts for approximately 10% of all cells (Supplementary Table 3), may be responsible for the trace incorporation of ^13^C into the CL-derived BP0 during the incubation (Figure 5). Another candidate for ^13^C-label incorporation from leucine is Thermoplasmatales, which occupy up to 4.9% of the total population in the Guaymas Basin cultures (Supplementary Table 3). The members of the Thermoplasmatales have been cultured with yeast extract as their carbon and energy sources (Itoh et al., 2007). They, therefore, may also be candidates for using leucine or its metabolized derivatives in the AOM enrichments. Lokiarchaeota, the third potential group, accounted for 0.4% of the whole population. Lokiarchaeota were only recently isolated and able to degrade amino acids *via* syntrophy, and they are likely to produce both archaeol and GDGTs as lipid membrane constituents (Imachi et al., 2020), which would be consistent with our study here. In summary, the low ^13^C labeling of archaeal lipids indicates that ancillary archaea play a small role in leucine turnover. However, given the widespread distribution of archaea and their postulated advantage over bacteria under conditions of severe energy stress, an archaeal contribution to the utilization of AOM-derived (dissolved) organic matter in the methane-laden sediments has to be taken into account (Biddle et al., 2006; Kubo et al., 2012; Yoshinaga et al., 2015).

## Conclusion

Meso- and thermophilic AOM cultures from the Guaymas Basin were incubated with position-specifically labeled ^13^C-leu to investigate heterotrophic lipid formation by ancillary community members. Most of the ^13^C from leucine was incorporated into branched-chain and unsaturated FAs of heterotrophic bacteria, such as Anaerolineae or Spirochetes. No ^13^C-leu incorporation into FAs was observed for the cultured *Ca. D. auxilii* SRB representative, confirming that this partner bacterium is an autotroph. Combining our results with former environmental information of FA patterns of different AOM consortia indicates that bacterial heterotrophs thrive on ^13^C-depleted AOM necromass or cell exudates/lysates in the form of amino acids in the marine environment, addressing the frequently observed strong decline of AOM biomass and lipid biomarkers below current SMTZs. In addition, archaeol-based IPLs and some tetraether CLs showed minor methane-independent assimilation of ^13^C, suggesting that ancillary, potentially heterotrophic archaea, such as Bathyarchaeota, Thermoplasmatales, and Lokiarchaeota, are active. All these taxa are minor community members in our enrichment cultures but commonly appear in subsurface sediments and can thus be specialists for the recycling of necromass in anoxic hydrocarbon-rich habitats. The AOM cultures, with their limited microbial diversity, appear to be a promising source of materials for confirming the function of these mostly uncultured microorganisms through targeted cultivation.

## Data Availability Statement

The original contributions presented in this study are included in the article/Supplementary Material, further inquiries can be directed to the corresponding author/s.

## Author Contributions

ME and Q-ZZ designed the research. Q-ZZ and GW performed the experiment. Q-ZZ analyzed the lipid data. Q-ZZ, ME, GW, and K-UH contributed to the discussion of the results and wrote the manuscript. All authors contributed to the article and approved the submitted version.

## Conflict of Interest

The authors declare that the research was conducted in the absence of any commercial or financial relationships that could be construed as a potential conflict of interest. The handling editor SE declared a past co-authorship with one of the authors, GW.

## Publisher’s Note

All claims expressed in this article are solely those of the authors and do not necessarily represent those of their affiliated organizations, or those of the publisher, the editors and the reviewers. Any product that may be evaluated in this article, or claim that may be made by its manufacturer, is not guaranteed or endorsed by the publisher.
