# Supplementary material for: Activity of Ancillary Heterotrophic Community Members in Anaerobic Methane-Oxidizing Cultures
Source: Front Microbiol. 2022 Jun 2;13:912299. doi: 10.3389/fmicb.2022.912299 (PMC9201399; doi:10.3389/fmicb.2022.912299)
Supplement: Supplementary file 1 [file Table_1.DOCX]

Table S1. Temporal development of carbon isotopic compositions of bacterial fatty acids (in ‰) compared to DIC in AOM enrichments from the Guaymas Basin at 37°C and 50°C (AOM37 and 50, respectively) over 28 days and the cultured bacterial partner Ca. D. auxilii over 40 days using different incubation conditions. n.a.: not available due to low signal during isotope analysis.

| Sample | Experiment: condition | Time (days) | DIC | C_14_ | *i*C_15_ | *ai*C_15_ | *i*C_16_ | C_16:1ω7_ | C_16_ | *i*C_17_ | *ai*C_17_ | C_17:1ω6_ | C_17_ | C_18:1ω9_ | C_18:1ω7_ | C_18_ |
| --- | --- | --- | --- | --- | --- | --- | --- | --- | --- | --- | --- | --- | --- | --- | --- | --- |
| AOM37 | 1: + CH_4_ + ^13^C-leu | 0 | -15 | -24 | -49 | -46 | -51 | -55 | -38 | -59 | -61 | -68 | n.a | -19 | -63 | -30 |
|  |  | 0.5 | -14 | -27 | 97 | 230 | 58 | -46 | -33 | 260 | 47 | -53 | -51 | 48 | -52 | -30 |
|  |  | 3 | -10 | -18 | 600 | 950 | 230 | -28 | -27 | 810 | 160 | -16 | 61 | 320 | -10 | -28 |
|  |  | 7 | 1 | -10 | 930 | 1300 | 300 | -8 | -19 | 1300 | 250 | 4 | 250 | 760 | 50 | -22 |
|  |  | 14 | 18 | 3 | 1700 | 2100 | 530 | -2 | -11 | 1800 | 340 | 18 | 430 | 1600 | 98 | -15 |
|  |  | 28 | 51 | 22 | 2300 | 2800 | 730 | 31 | -1 | 2300 | 400 | 51 | 700 | 2200 | 120 | -12 |
|  | 2: + CH_4_ | 28 | -20 | -25 | -50 | -46 | -52 | -55 | -38 | -59 | -61 | -68 | n.a | -17 | -63 | -31 |
|  | 3: + ^13^C-leu | 28 | 68 | 24 | 1600 | 2800 | 840 | -88 | -17 | 1800 | 330 | n.a | 410 | 2300 | 82 | -13 |
|  |  |  |  |  |  |  |  |  |  |  |  |  |  |  |  |  |
| AOM50 | 1: + CH_4_ + ^13^C-leu | 0 | -26 | -25 | n.a | -26 | -45 | -27 | -28 | n.a | -45 | n.a | -27 | -25 | -35 | -28 |
|  |  | 28 | 113 | 21 | 6400 | 1300 | 590 | 180 | 5 | 3900 | -21 | 51 | 470 | 130 | 110 | -20 |
|  | 2: + CH_4_ | 28 | -34 | -25 | n.a | -26 | -45 | -27 | -28 | n.a | -46 | n.a | -27 | -25 | -36 | -28 |
|  | 3: + ^13^C-leu | 28 | 125 | -19 | 5700 | 970 | n.a | -26 | -23 | 3700 | 170 | n.a | 230 | 14 | n.a | -29 |
|  |  |  |  |  |  |  |  |  |  |  |  |  |  |  |  |  |
| *Ca.* D. auxilii | 4: + H_2_ + ^13^C-leu | 0 | -18 | -25 | n.a. | n.a. | n.a | -28 | -24 | n.a. | n.a. | n.a | -23 | n.a. | -27 | -22 |
|  |  | 40 | -18 | -26 | n.a. | n.a. | n.a | n.a | -26 | n.a. | n.a. | n.a | -24 | n.a. | -28 | -24 |

Table S2. Temporal development of carbon isotopic compositions of archaeal lipids (in ‰), separated into core and intact polar lipids (CL and IPL, respectively), compared to DIC in AOM enrichments from the Guaymas Basin at 37°C and 50°C (AOM37 and 50, respectively) over 28 days using different incubation conditions. n.a.: not available due to low signal during isotope analysis.

| Sample | Experiment: condition | Time (days) | DIC | CL-Phy | CL-BP0 | CL-BP1 | CL-BP2 | IPL-Phy | IPL-BP0 | IPL-BP1 | IPL-BP2 |
| --- | --- | --- | --- | --- | --- | --- | --- | --- | --- | --- | --- |
| AOM37 | 1: + CH_4_ + ^13^C-leu | 0 | -15 | -72 | -71 | -73 | -72 | -71 | -70 | -71 | -68 |
|  |  | 0.5 | -14 | -71 | -71 | -72 | -71 | -60 | n.a | n.a | n.a |
|  |  | 3 | -10 | -66 | -71 | -72 | -71 | -48 | -67 | -68 | -68 |
|  |  | 7 | 1 | -57 | -71 | -73 | -71 | -43 | n.a | n.a | n.a |
|  |  | 14 | 18 | -26 | -70 | -74 | -71 | -5 | -63 | -66 | -68 |
|  |  | 28 | 51 | -1 | -68 | -73 | -71 | 42 | -67 | -70 | -68 |
|  | 2: + CH_4_ | 28 | -20 | -72 | -71 | -73 | -72 | -71 | -70 | -72 | -68 |
|  | 3: + ^13^C-leu | 28 | 68 | -22 | -66 | -73 | -71 | 47 | -66 | -70 | -68 |
|  |  |  |  |  |  |  |  |  |  |  |  |
| AOM50 | 1: + CH_4_ + ^13^C-leu | 0 | -26 | -74 | -71 | -70 | -70 | n.a | n.a | n.a | n.a |
|  |  | 28 | 113 | -7 | -55 | -70 | -68 | n.a | n.a | n.a | n.a |
|  | 2: + CH_4_ | 28 | -34 | -74 | -71 | -71 | -70 | n.a | n.a | n.a | n.a |
|  | 3: + ^13^C-leu | 28 | 125 | -23 | -65 | -69 | -68 | n.a | n.a | n.a | n.a |

Table S3. Classification of metagenomic 16S rRNA gene fragments on phylogenetic levels of Class (normalized to 100%) in AOM37 and AOM60 (data from Krukenberg et al., 2018) as well as their dominant C assimilation pathway and signature lipids based on published literature or this study. The composition of the AOM60 culture is largely identical to the tested AOM50 culture. Both cultures contain the same ANME-1 and HotSeep-1 strains, and the cultures show similar activity at both temperatures.

| **Name** | **AOM37** | **AOM60** | **C assimilation** | **Signature lipids** |
| --- | --- | --- | --- | --- |
| **Archaea** |  |  |  |  |
| ANME-1 | 56.3 | 39.8 | autotrophic | GDGT-0, -1, -2 (**A**) |
| Thermoplasmata | 0.9 | 4.9 | heterotrophic | Archaeol, GDGT-0 (**B**) |
| Lokiarchaeota | 0.4 |  | heterotrophic | Archaeol, GDGT-0, 1, 2 (**C**) |
| Bathyarchaeota | | 10.5 | heterotrophic | Archaeol, GDGT-0, -1, -2 (**D**) |
| Marine Hydrothermal Vent Group 1  (MHVG-1)_unclassified | | 0.7 |  |  |
| Archaeoglobi | | 0.7 |  |  |
| Thermococci | | 0.4 |  |  |
| Thermoprotei | | 0.3 |  |  |
| Euryarchaeota_unclassified | | 0.3 |  |  |
| Korarchaeota Incertae Sedis | | 0.3 |  |  |
| Terrestrial Hot Spring Group (THSCG) | | 0.3 |  |  |
| **Bacteria** |  |  |  |  |
| Deltaproteobacteria_SEEP-SRB2 | 25.6 |  | autotrophic | C_16:0_, C_18:1ω7_, C_18:0_ (**E**) |
| Deltaproteobacteria_Ca. D. auxilii | | 13.7 | autotrophic | C_16:0_, C_18:0_ (**E**) |
| Deltaproteobacteria_others | 2.6 | 10.4 |  |  |
| Deferribacteres | 1.2 |  |  | |
| Spirochaetes | 5.0 |  | heterotrophic | *i*C_15_, *i*C_17_, C_18:1ω7_ (**F**) |
| Chloroflexi_unclassified | 0.2 | 0.8 | heterotrophic |  |
| Anaerolineae | 2.2 | 3.7 | heterotrophic | *i*C_15_, *i*C_17_, C_16:0_ (**G**) |
| Phycisphaerae | | 1.5 | heterotrophic | C_16:0_, *i*C_16_, *i*C_16:1_ (**H**) |
| Candidate division JS1_unclassified | 0.7 |  |  |  |
| Candidate division WS3_unclassified | 0.6 |  |  |  |
| Candidate division KB1_unclassified | 0.2 | 1.8 |  |  |
| Candidate division OP3_unclassified | 0.3 |  |  |  |
| Candidate division OP8_unclassified | | 0.3 |  |  |
| Thermotogae | 0.2 | 0.6 |  |  |
| AK8 |  | 0.4 |  |  |
| Proteobacteria_unclassified | | 1.6 |  |  |
| Synergistia | 0.2 |  |  |  |
| **Unclassified** | 2 | 6.5 |  |  |

**A**: (Schouten et al., 2001; Blumenberg et al., 2004; Elvert et al., 2005); **B**: (Yoshinaga et al., 2015; Yin et al., 2022); **C**: (Imachi et al., 2020); **D**: (Yu et al., 2018); **E**: (this study); **F**: (Livermore and Johnson, 1974; Vishnuvardhan Reddy et al., 2013); **G**: (Yamada et al., 2006); **H**: (Fukunaga et al., 2009)


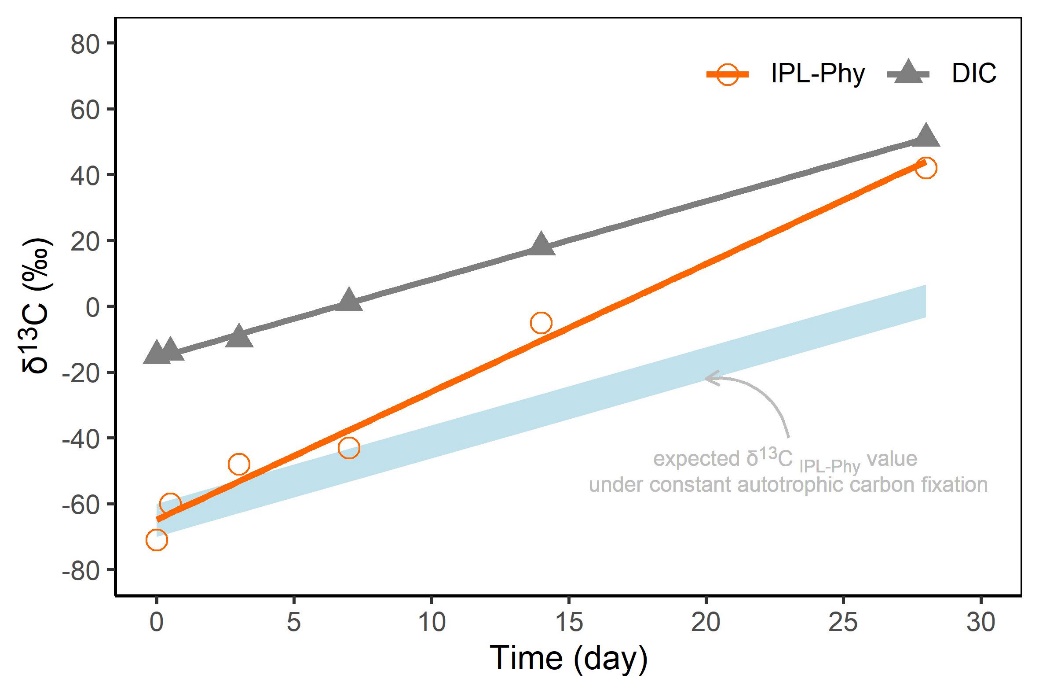


Fig. S1. Development of δ^13^C values (in ‰) of DIC and IPL-Phy during incubation of AOM37 over 28 days in experiment 1. The stronger increasing δ^13^C values of IPL-Phy opposed to DIC indicate a contribution of carbon used for archaeal lipid synthesis being different to DIC. The expected offset is displayed by the light blue bar assuming constant isotopic fractionation during autotrophic carbon fixation of the ANMEs.

**References**

Blumenberg, M., Seifert, R., Reitner, J., Pape, T., and Michaelis, W. (2004). Membrane lipid patterns typify distinct anaerobic methanotrophic consortia. *PNAS* 101, 11111–11116. doi:10.1073/pnas.0401188101.

Elvert, M., Hopmans, E. C., Treude, T., Boetius, A., and Suess, E. (2005). Spatial variations of methanotrophic consortia at cold methane seeps: implications from a high-resolution molecular and isotopic approach. *Geobiology* 3, 195–209. doi:https://doi.org/10.1111/j.1472-4669.2005.00051.x.

Fukunaga, Y., Kurahashi, M., Sakiyama, Y., Ohuchi, M., Yokota, A., and Harayama, S. (2009). Phycisphaera mikurensis gen. nov., sp. nov., isolated from a marine alga, and proposal of Phycisphaeraceae fam. nov., Phycisphaerales ord. nov. and Phycisphaerae classis nov. in the phylum Planctomycetes. *The Journal of General and Applied Microbiology* 55, 267–275. doi:10.2323/jgam.55.267.

Imachi, H., Nobu, M. K., Nakahara, N., Morono, Y., Ogawara, M., Takaki, Y., et al. (2020). Isolation of an archaeon at the prokaryote–eukaryote interface. *Nature* 577, 519–525. doi:10.1038/s41586-019-1916-6.

Krukenberg, V., Riedel, D., Gruber-Vodicka, H. R., Buttigieg, P. L., Tegetmeyer, H. E., Boetius, A., et al. (2018). Gene expression and ultrastructure of meso- and thermophilic methanotrophic consortia. *Environ. Microbiol.* 20, 1651–1666. doi:10.1111/1462-2920.14077.

Livermore, B. P., and Johnson, R. C. (1974). Lipids of the Spirochaetales: comparison of the lipids of several members of the genera Spirochaeta, Treponema, and Leptospira. *J Bacteriol* 120, 1268–1273. Available at: https://www.ncbi.nlm.nih.gov/pmc/articles/PMC245910/ [Accessed January 19, 2022].

Schouten, S., Wakeham, S. G., and Sinninghe Damsté, J. S. (2001). Evidence for anaerobic methane oxidation by archaea in euxinic waters of the Black Sea. *Organic Geochemistry* 32, 1277–1281. doi:10.1016/S0146-6380(01)00110-3.

Vishnuvardhan Reddy, S., Aspana, S., Tushar, D. L., Sasikala, Ch., and Ramana, Ch. (2013). Spirochaeta sphaeroplastigenens sp. nov., a halo-alkaliphilic, obligately anaerobic spirochaete isolated from soda lake Lonar. *Int. J. Syst. Evol. Microbiol* 63, 2223–2228. doi:10.1099/ijs.0.046292-0.

Yamada, T., Sekiguchi, Y., Hanada, S., Imachi, H., Ohashi, A., Harada, H., et al. (2006). Anaerolinea thermolimosa sp. nov., Levilinea saccharolytica gen. nov., sp. nov. and Leptolinea tardivitalis gen. nov., sp. nov., novel filamentous anaerobes, and description of the new classes Anaerolineae classis nov. and Caldilineae classis nov. in the bacterial phylum Chloroflexi. *Int. J. Syst. Evol. Microbiol* 56, 1331–1340. Available at: https://www.microbiologyresearch.org/content/journal/ijsem/10.1099/ijs.0.64169-0.

Yin, X., Zhou, G., Cai, M., Zhu, Q.-Z., Richter-Heitmann, T., Aromokeye, D. A., et al. (2022). Catabolic protein degradation in marine sediments confined to distinct archaea. *ISME J*, 1–10. doi:10.1038/s41396-022-01210-1.

Yoshinaga, M. Y., Lazar, C. S., Elvert, M., Lin, Y.-S., Zhu, C., Heuer, V. B., et al. (2015). Possible roles of uncultured archaea in carbon cycling in methane-seep sediments. *Geochimica et Cosmochimica Acta* 164, 35–52. doi:10.1016/j.gca.2015.05.003.

Yu, T., Wu, W., Liang, W., Lever, M. A., Hinrichs, K.-U., and Wang, F. (2018). Growth of sedimentary Bathyarchaeota on lignin as an energy source. *PNAS* 115, 6022–6027. doi:10.1073/pnas.1718854115.
